# Incomplete Hippocampal Inversion: A Comprehensive MRI Study of Over 2000 Subjects

**DOI:** 10.3389/fnana.2015.00160

**Published:** 2015-12-22

**Authors:** Claire Cury, Roberto Toro, Fanny Cohen, Clara Fischer, Amel Mhaya, Jorge Samper-González, Dominique Hasboun, Jean-François Mangin, Tobias Banaschewski, Arun L. W. Bokde, Uli Bromberg, Christian Buechel, Anna Cattrell, Patricia Conrod, Herta Flor, Juergen Gallinat, Hugh Garavan, Penny Gowland, Andreas Heinz, Bernd Ittermann, Hervé Lemaitre, Jean-Luc Martinot, Frauke Nees, Marie-Laure Paillère Martinot, Dimitri P. Orfanos, Tomas Paus, Luise Poustka, Michael N. Smolka, Henrik Walter, Robert Whelan, Vincent Frouin, Gunter Schumann, Joan A. Glaunès, Olivier Colliot, The IMAGEN Consortium

**Affiliations:** ^1^Institut national de la santé et de la recherche médicale, U1127Paris, France; ^2^Centre National de la Recherche Scientifique, UMR 7225 Institut du Cerveau et de la Moelle épinièreParis, France; ^3^Sorbonne Universités, Université Pierre et Marie Curie Univ Paris 06, UMR S 1127Paris, France; ^4^Institut du Cerveau et de la Moelle épinière, Institut du Cerveau et de la Moelle épinièreParis, France; ^5^Inria, Aramis Team, Centre de Recherche Paris-RocquencourtParis, France; ^6^Centre d'Acquisition et de Traitement des ImagesParis, France; ^7^Centre National de la Recherche Scientifique, Genes, Synapses and Cognition, URA 2182, Institut PasteurParis, France; ^8^Human Genetics and Cognitive Functions, Institut PasteurParis, France; ^9^Institut d'Imagerie Biomédicale; Commissariat à l'énergie atomique et aux énergies alternatives; Direction des Sciences du VivantGif-Sur-Yvette, France; ^10^Departments of Neuroradiology and Neurology, AP-HP, Hôpital de la Pitié-SalpétrièreParis, France; ^11^Department of Child and Adolescent Psychiatry and Psychotherapy, Clinical Faculty Mannheim, Central Institute of Mental Health, University of HeidelbergMannheim, Germany; ^12^Discipline of Psychiatry, School of Medicine, Trinity College DublinDublin, Ireland; ^13^Institute of Neuroscience, Trinity College DublinDublin, Ireland; ^14^Department of Systems Neuroscience, Universitätsklinikum Hamburg EppendorfHamburg, Germany; ^15^Department of Psychology, Stanford UniversityStanford, CA, USA; ^16^Institute of Psychiatry, Psychology and Neuroscience, King's College LondonLondon, UK; ^17^MRC Social, Genetic and Developmental Psychiatry CentreLondon, UK; ^18^Département de Psychiatrie, Centre Hospitalier Universitaire Sainte-Justine, Université de MontrealMontreal, QC, Canada; ^19^Department of Cognitive and Clinical Neuroscience, Central Institute of Mental Health, Medical Faculty Mannheim, Heidelberg UniversityMannheim, Germany; ^20^Department of Psychiatry and Psychotherapy, Campus Charité Mitte, Charité–Universitätsmedizin BerlinGermany; ^21^School of Physics and Astronomy, University of NottinghamNottingham, UK; ^22^Physikalisch-Technische BundesanstaltBerlin, Germany; ^23^Institut national de la santé et de la recherche médicale U1000, Neuroimagerie en Psychiatrie, Université Paris-Sud, Université Paris DescartesParis, France; ^24^AP-HP, Department of Adolescent Psychopathology and Medicine, Maison de Solenn, Cochin Hospital, University Paris Descartes, Sorbonne Paris CitéParis, France; ^25^Rotman Research Institute, BaycrestToronto, ON, Canada; ^26^Departments of Psychology and Psychiatry, University of TorontoToronto, Canada; ^27^Center for Developing Brain, Child Mind InstituteNew York, NY, USA; ^28^Department of Child and Adolescent Psychiatry, Medical University of ViennaVienna, Austria; ^29^Department of Psychiatry and Neuroimaging Center, Technische Universität DresdenDresden, Germany; ^30^Berlin School of Mind and Brain, Humboldt University BerlinBerlin, Germany; ^31^Department of Psychology, University College DublinDublin, Ireland; ^32^MAP5, Université Paris Descartes, Sorbonne Paris CitéParis, France

**Keywords:** human hippocampus, malrotation, anatomical variability, brain development, cortical sulci, IMAGEN database, MRI, Large database

## Abstract

The incomplete-hippocampal-inversion (IHI), also known as malrotation, is an atypical anatomical pattern of the hippocampus, which has been reported in healthy subjects in different studies. However, extensive characterization of IHI in a large sample has not yet been performed. Furthermore, it is unclear whether IHI are restricted to the medial-temporal lobe or are associated with more extensive anatomical changes. Here, we studied the characteristics of IHI in a community-based sample of 2008 subjects of the IMAGEN database and their association with extra-hippocampal anatomical variations. The presence of IHI was assessed on T1-weighted anatomical magnetic resonance imaging (MRI) using visual criteria. We assessed the association of IHI with other anatomical changes throughout the brain using automatic morphometry of cortical sulci. We found that IHI were much more frequent in the left hippocampus (left: 17%, right: 6%, χ^2^−*test*, *p* < 10^−28^). Compared to subjects without IHI, subjects with IHI displayed morphological changes in several sulci located mainly in the limbic lobe. Our results demonstrate that IHI are a common left-sided phenomenon in normal subjects and that they are associated with morphological changes outside the medial temporal lobe.

## Introduction

The incomplete hippocampal inversion (IHI) is an atypical anatomical pattern of the hippocampus which prominent features are a round, verticalized, and medially positioned hippocampus and a deep collateral sulcus (Baulac et al., 1998; Bernasconi et al., 2005). Different terms have been used to refer to this atypical pattern including “incomplete hippocampal inversion” (Bajic et al., 2008; Raininko and Bajic, 2010), “hippocampal malrotation” (Barsi et al., 2000; Peltier et al., 2005; Gamss et al., 2009), “abnormal hippocampal formation” (Bernasconi et al., 2005), “developmental changes of the hippocampal formation” (Baulac et al., 1998). IHI were mostly described in patients with epilepsy, in particular in patients with malformations of cortical development (MCD) but also in temporal lobe epilepsy (TLE; Lehéricy et al., 1995; Baulac et al., 1998; Bernasconi et al., 2005; Bajic et al., 2009), with a prevalence of 30–50%. IHI are not specific of epilepsy and have also been reported in healthy subjects, although with an apparently lower frequency (Bronen and Cheung, 1991; Bernasconi et al., 2005; Bajic et al., 2008). This has led to speculate that IHI may be the end of the phenotypic spectrum of normal hippocampal shape (Bernasconi et al., 2005). IHI are thought to be of developmental origin, as shown by studies in neonates (Righini et al., 2006; Raininko and Bajic, 2010). It is thus tempting to speculate that IHI may be a marker of atypical brain development.

The anatomical pattern of incomplete inversion may be factor of susceptibility to pathological processes. The high prevalence of IHI in patients with epilepsy and MCD has led to think that they may be a marker of abnormal development. Furthermore, IHI have been noted in association with different developmental defects, including agenesis of the corpus callosum (Atlas et al., 1986), and patients with genetic anomalies (Fitoz et al., 2003; Grosso et al., 2003; Andrade et al., 2013; Boronat et al., 2015) that present with increased risk of neuropsychiatric disorders including autism spectrum disorders (Campbell et al., 2006) and schizophrenia (Baker et al., 2011). However, in order to study IHI as a marker of abnormal development in neuropsychiatric diseases, it is important to first fully characterize them in the normal population.

IHI can also challenge the performance of automatic hippocampal segmentation methods, lower segmentation accuracy being found in the presence of IHI (Kim et al., 2012a). While multi-template approaches appear more robust to the presence of IHI than other types of approaches (Kim et al., 2012b), it remains important to adequately characterize IHI to ensure that volumetry or morphometry studies are not biased by their occurrence.

Until now, IHI in normal subjects remain insufficiently characterized. First, the prevalence of IHI in normal subjects is a matter of debate (Gamss et al., 2009; Raininko and Bajic, 2010). Some authors consider IHI a common finding in healthy subjects (Bajic et al., 2008; Raininko and Bajic, 2010) while other report that they are a rare pattern (Gamss et al., 2009). A possible reason for these discrepancies is that previous studies of IHI in subjects without epilepsy have included a small number of healthy subjects (Bernasconi et al., 2005; Bajic et al., 2008) or have included patients without epileptic seizures but referred for other neurological conditions (Bajic et al., 2008; Gamss et al., 2009), thus leading to an imprecise estimation of their prevalence. Moreover, a probable lateralization of IHI, predominantly in the left hemisphere, has been noted (Baulac et al., 1998; Bernasconi et al., 2005; Raininko and Bajic, 2010). Finally, it is unknown whether this unusual pattern is confined to the medial temporal lobe or is associated with more widespread morphological changes throughout the brain.

Our purpose was to study the prevalence and characteristics of IHI in a large population of normal subjects. We studied a community-based sample of 2008 young subjects of the European database IMAGEN (Schumann et al., 2010). The presence of IHI was assessed visually on 3D T1-weighted magnetic resonance imaging (MRI) data. To that aim, we designed a new visual scale of IHI that includes the most representative published criteria of IHI (Baulac et al., 1998; Bernasconi et al., 2005), includes a reasonable number of items and leads to a robust assessment. In order to explore the association of IHI with extra-hippocampal changes, we performed a morphometric analysis of 45 cortical sulci in each hemisphere, which were extracted using automatic image processing software.

## Materials and methods

### Participants and MRI data

We studied a community-based sample of young subjects from the multi-centric European database IMAGEN (Schumann et al., 2010; http://www.imagen-europe.com/). Local ethics committee^1^ approved the study. Participants' parents gave informed written consent, and the adolescents gave written assent. We studied 2089 subjects with high-resolution T1-weighted anatomical MRI. For all subjects, T1-weighted MRI were acquired on 3 Tesla scanners (Siemens Verio and TimTrio, Philips Achieva, General Electric Signa Excite, and Signa HDx) using a 3D Magnetization Prepared Rapid Acquisition Gradient Echo (MPRAGE) sequence (*TR* = 2300 ms; *TE* = 2.8 ms, flip angle = 9°; resolution: 1 × 1 × 1 mm^3^). We performed quality control of the MRI data, checking for general quality as well as specific visibility of the hippocampal formations. The MRI was judged of sufficient quality for assessment of IHI for 2008 subjects, which were entered into the study (characteristics of the subjects are given in Table 1). In order to perform IHI assessment with a standardized orientation, T1-weighted MRIs were then registered toward the MNI152 atlas using the FSL software using the fully automated affine transformation FLIRT (Jenkinson and Smith, 2001; Jenkinson et al., 2002).

**Table 1:** **Characteristics of the studied population**.

**Number of subjects**	**Gender**	**Age in years mean ± SD (range)**	**Handedness (Right/Left/Both)**
2008	1029 F/978 M	14.5 ± 0.4 (12.9–17.2)	1740/218/14

F, Female; M, Male; SD, Standard-Deviation.

### Criteria of incomplete hippocampal inversions

A round and verticalized hippocampus, a deep collateral sulcus, and a medial positioning globally characterize an IHI. For rating IHI, five individual criteria (named C1 to C5) and a global criterion named C0 were defined.

#### Criterion C1: verticality and roundness of the hippocampal body

Criterion C1 assesses both the roundness of the hippocampal body and its verticality. Some studies have considered roundness and verticality simultaneously (Lehéricy et al., 1995; Bernasconi et al., 2005), while others have considered them separately (Baulac et al., 1998) or have considered only the roundness (Barsi et al., 2000; Bajic et al., 2008; Gamss et al., 2009; Stiers et al., 2010). In our case, we considered roundness and verticality as a single criterion, in order to limit the number of criteria.

**Figure 1:**
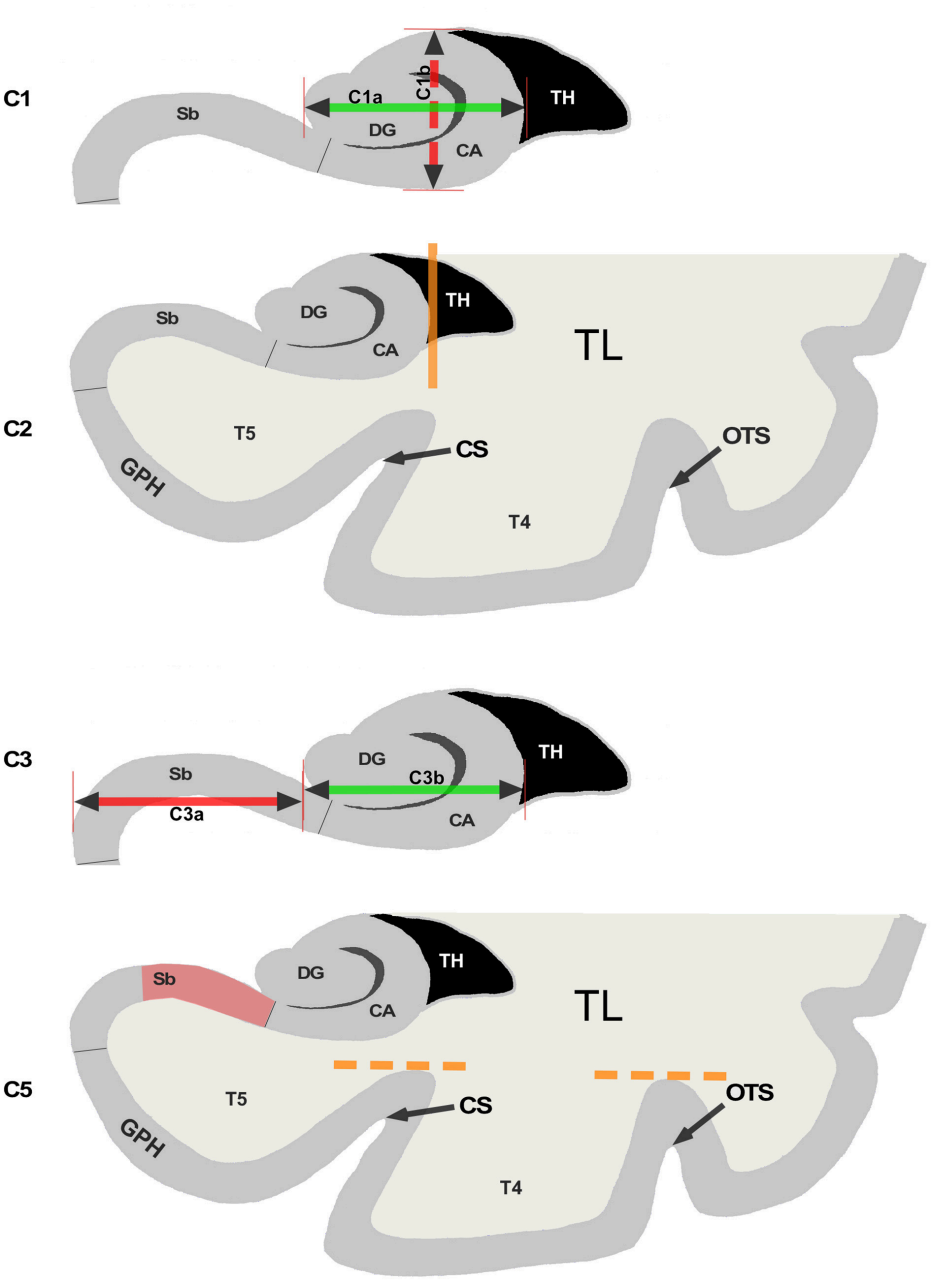
**Illustration of the 5 criteria used for the evaluation of Incomplete Hippocampal Inversions**. C1: Roundness and verticality. The horizontal arrow (C1a) goes from the medial part of the Dentate Gyrus (DG) to the lateral part of the hippocampus. The vertical arrow (C1b) goes from the bottom to the top part of the Cornus Ammonis (CA) C2: Verticality and depth of the collateral sulcus. The vertical line indicates the lateral border of the hippocampus which is used to determine if the sulcus is deep or not. CS indicates the collateral sulcus and OTS the occipito temporal sulcus. C3: Medial positioning. The C3a segment indicates the part of the subiculum (Sb) not covered by the DG. The C3b segment indicates the part of CA covered by the DG. C5: Orientation of the sulci of the fusiform gyrus. The dotted lines indicate the top of the sulci CS and OTS. The upper part of the subiculum is the red area.

Criterion C1 was evaluated on the first half of the hippocampal body, on coronal slices. The principles used to evaluate this criterion are illustrated on Figure 1C1. Two segments *C1a* and *C1b* are determined. Segment *C1a* represents the width of the hippocampus in a coronal view. It is estimated parallel to the ventral part of the cornu Ammonis (CA)/subiculum and goes from the medial part of the dentate gyrus to the lateral part of CA. The segment C1b in a coronal view represents the height of the hippocampal body. *C1b* must be perpendicular to segment *C1a* and goes from the dorsal part of the hippocampus to the ventral part of CA. The roundness is evaluated on three levels: flat (width larger than height, i.e., *C1a* > *C1b*), round (*C1a* = *C1b*) or oval (C1a < C1b). For the verticality, three levels were used: horizontal if the segment *C1a* is horizontal (with a tolerance of around 10°), oblique if C1a is neither horizontal nor vertical (around 45°) and vertical if segment *C1a* is vertical with also a tolerance of around 10°.

Segments on Figure 1 are here to illustrate and help the new observer to understand the criterion. The evaluation of the MRI is made without tracing such segments.

**Figure 2:**
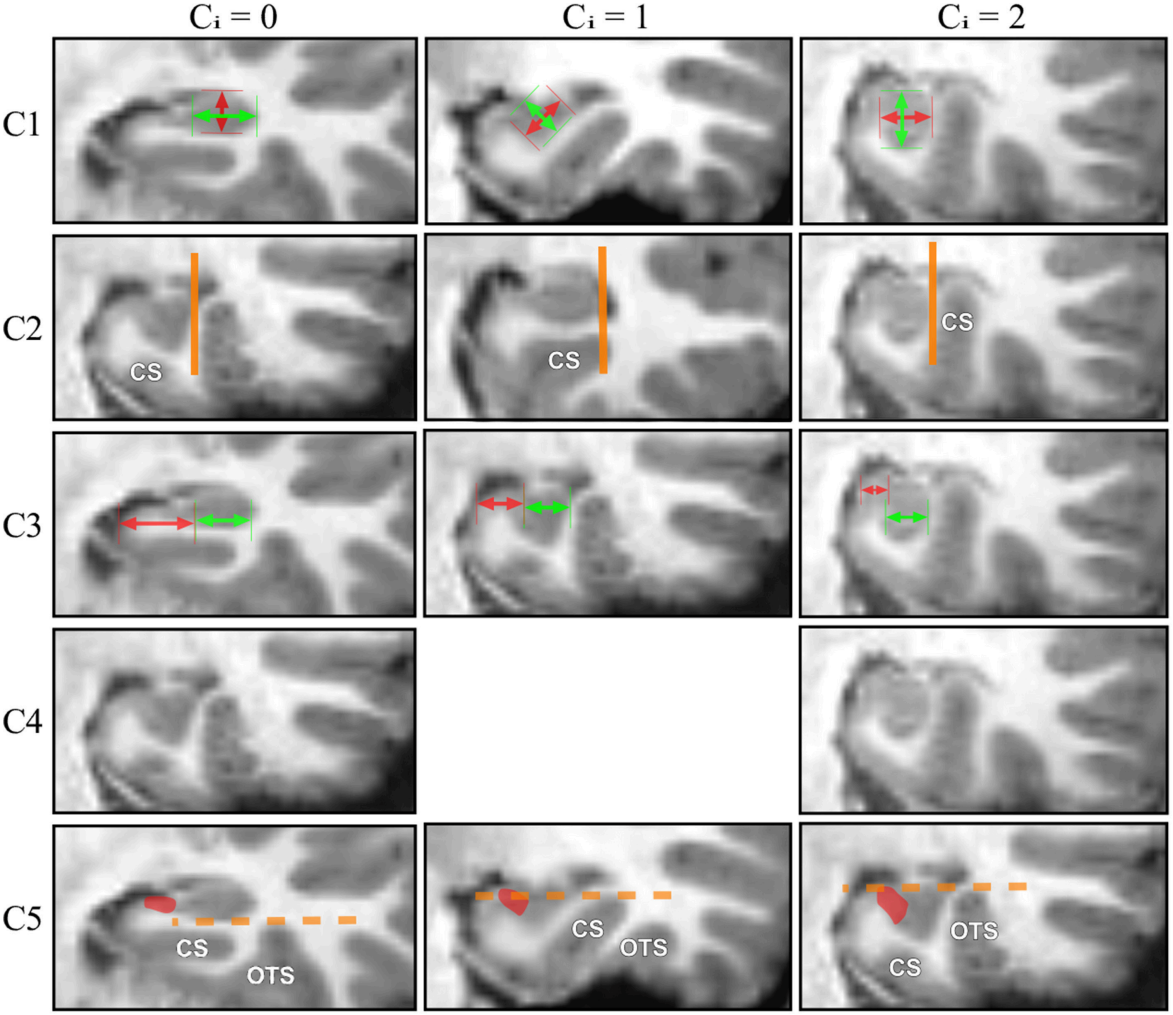
**For each IHI criterion, examples corresponding to 3 different grades (0, 1, 2) are displayed**.

When roundness and verticality have been determined, they are reported to determine the grade for the C1 criterion following the rules defined in Table 2. Examples are shown on Figure 2, basically, a flat horizontal hippocampus has a grade C1 = 0, a round hippocampus has a grade C1 = 1, and a vertical hippocampus has a grade C1 = 2.

**Table 2:** **Evaluation of the criterion C1, based on the verticality and roundness of the hippocampal body in a coronal view**.

**Roundness/verticality**	**Horizontal**	**Oblique**	**Vertical**
Flat	0	0.5	NA
Round	0.5	1	2
Oval	1	1.5	2

NA, not applicable. Grades are between 0 and 2.

#### Criterion C2: collateral sulcus

This criterion assesses the verticality and depth of the collateral sulcus relatively to the size of the hippocampus. The collateral sulcus separates the fourth (T4) from the fifth convolution (T5) of the temporal lobe, and supports the collateral eminence. This criterion is evaluated at the level of the hippocampal body, where the collateral sulcus is easier to identify.

In Figure 1C2, the vertical orange line indicates the lateral limit of the hippocampus. The evaluation of this criterion has been defined as follows: if the collateral sulcus (CS) does not cross the lateral limit of the hippocampus, the grade for C2 will be from 0 to 1, i.e., 0, 0.5, or 1. If the CS crosses the lateral limit of the hippocampus, the grade will be from 1 to 2 (i.e., 1, 1.5, or 2). A sulcus can be horizontal, oblique or vertical, a more vertical sulcus will result in a higher grade, as presented in Table 3. Examples are given on Figure 2.

**Table 3:** **Evaluation of the criterion C2, based on the collateral sulcus**.

	**CS**<**H**		**CS** = **H**		**CS** > **H**		
Verticality	hor	obl/ver	hor/obl	ver	hor	obl	ver
Grade	0	0.5	1	1.5	1	1.5	2

CS, collateral Sulcus; H, Hippocampus; hor, horizontal; obl, oblique; ver, vertical. The depth of the collateral sulcus (CS) is defined by its length compared to the width of the hippocampus (H). The verticality is evaluated on three levels: horizontal (hor) oblique (obl) and vertical (ver). Grades are between 0 and 2.

#### Criterion C3: medial positioning

This criterion assesses the medial positioning of the hippocampus. To evaluate this criterion, we consider the length of the part of the subiculum that is not covered by the dentate gyrus (segment *C3a* on Figure 1C3) relatively to the ventral part of CA/subiculum that is covered by the dentate gyrus (segment *C3b* on Figure 1C3). Even if the hippocampus is vertical or oblique, segments *C3b* and *C3a* are defined orthogonally to the brain midline. In addition, we considered whether the temporal horn (TH) of the lateral ventricle was empty or filled by CSF.

The evaluation is made on five levels: from 0 for a very lateral positioning to 2 for a very medial one. Evaluations are given in Table 4. The two extreme grades are without considering the TH: if the *C3a* segment is not visible because the hippocampus is too close to the ambient cistern, the grade is 2. Similarly, if the subiculum is very long compared to the *C3b* part, the grade is 0, regardless of the TH. In other situations, the grade is modulated by the configuration of the TH, as presented in Table 4. Examples are displayed on Figure 2.

**Table 4:** **Evaluation of the criterion C3, based on the medial positioning of the hippocampus in a coronal view**.

	**C3a < < C3b**	**C3a < C3b**	**C3a = C3b**	**C3a > C3b**	**C3a >> C3b**
TH emptied	2	1	0.5	0	0
TH filled	2	1.5	1	0.5	0

C3a, Subiculum part not covered by the dentate gyrus; C3b, Cornus Ammonis part covered by the dentate gyrus; TH, Temporal Horn of the lateral ventricle. The medial positioning of the hippocampus is determined by comparing the length of the subiculum part (Sp) not covered by the dentate gyrus to the length of the CA part (CAp) covered by the dentate gyrus. Grades are between 0 and 2.

#### Criterion C4: subiculum

This criterion assesses the thickness of the subiculum, as in Bernasconi et al. (2005). The subiculum is considered as abnormal if it is bulging upward, therefore looking thickened, which corresponds to a grade equal to 2. Otherwise, the subiculum is considered normal and the grade is 0.

#### Criterion C5: sulci of the fusiform gyrus (T4)

This is a new criterion, which complements criterion C2. Indeed, we observed that IHI are not only associated with atypical patterns of the collateral sulcus (CS) but also of the occipito-temporal sulcus (OTS) which separates the fourth temporal (T4) and the third temporal (T3) convolutions. In that case, the OTS is deep and comes laterally to the hippocampus. Criterion C5 takes into account both the CS and the OTS. We evaluate if one of these sulci is deep enough to cross the level of the subiculum. In Figure 1C5, we can see that none of the two sulci, which superior parts are indicated by dotted lines, goes over the subiculum indicated by the red area.

The evaluation is made on three levels. If none of the sulci exceed the level of the subiculum, the grade is 0. If one of the sulci crosses sidewise the level of the subiculum, with an oblique orientation, the grade is 1; if a sulcus exceeds vertically the subiculum, the grade is 2. Examples are displayed on Figure 2.

### Criterion C0: global aspect of the hippocampus

In addition to these five individual criteria, we also defined a global criterion indicating the presence of an IHI. This was done in order to provide a global assessment of the presence of an IHI. Criterion C0 is evaluated on three levels, a grade of 0 is given if the hippocampus has a common aspect, a grade of 2 is given if the hippocampus has a pronounced incomplete inversion which corresponds to the total IHI in the literature (Baulac et al., 1998; Bajic et al., 2008), and a 1 is given if the hippocampus does not have a common aspect (flat and horizontal) neither a clear incomplete inversion, which corresponds to a partial IHI (Bajic et al., 2008).

### Assessment of incomplete hippocampal inversions

IHI were assessed by two trained observers (CC and FC). Forty-two subjects were randomly selected to evaluate the intra- and inter-observer reproducibility of the evaluations. Half of the remaining 1966 subjects were evaluated by CC; the other half was evaluated by FC. Additionally CC checked the evaluations given by FC, and FC checked the evaluations given by CC in order to homogenize evaluations.

### Intra- and inter-observer reproducibility

To evaluate the reproducibility, each observer (CC and FC) evaluated twice the same series of 42 subjects. The first time was after the evaluation of 200 subjects, and the second time after assessment of 900 subjects, with at least 3 weeks in between. Intra- and inter-observer reproducibility were computed using kappa tests (Viera and Garrett, 2005) for the criteria C0, C4, and C5, and a weighted kappa tests for criteria C1, C2, and C3. Reproducibility of criterion C4 could not be evaluated because all subjects used for reproducibility assessment had a C4 grade equal to zero.

### Sulcal morphometry

**Figure 3:**
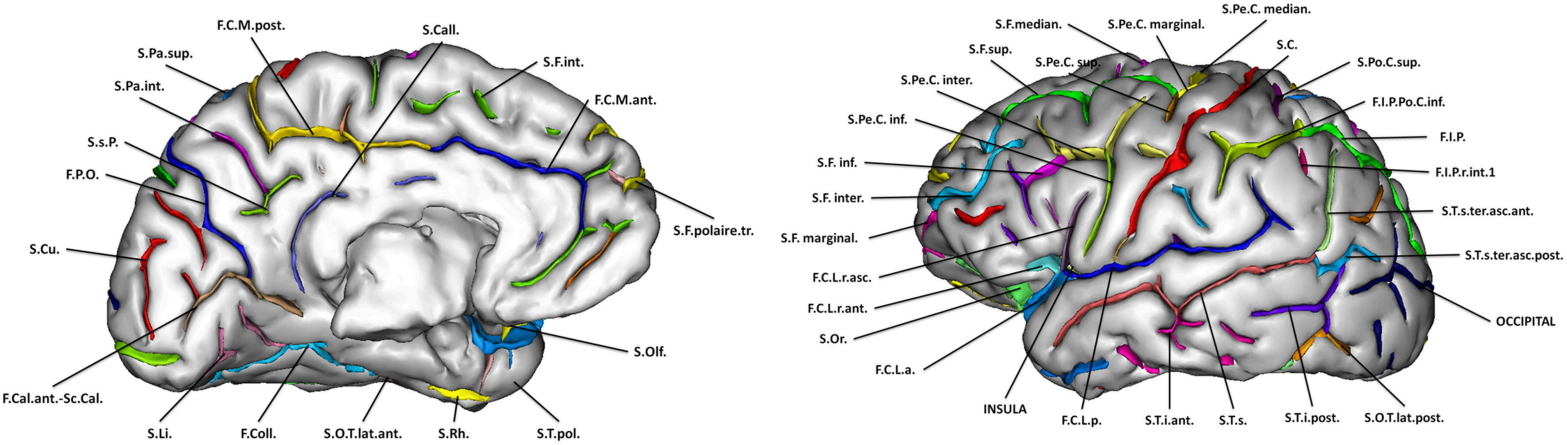
**Sulci segmented via the Morphologist toolbox of the Brainvisa software**.

For each subject, cortical sulci were automatically extracted and identified from T1-weighted MRI using the Morphologist toolbox (Fischer et al., 2012) of the BrainVisa software (http://brainvisa.info). Briefly, this method involves the following steps: (1) brain extraction and separation of hemispheres; (2) classification of white matter (WM), gray matter (GM), and cerebro-spinal fluid (CSF); (3) reconstruction of the surfaces corresponding to the GM-WM and GM-CSF interface maps; (4) extraction of the sulcal folds by segmenting the skeletonized GM/CSF interface; (5) automatic labeling of individual cortical sulci using a machine learning approach. We analyzed 45 cortical sulci per hemisphere. These sulci are listed on Figure 3.

In all subjects, the output of the automatic procedure was quality controlled by a trained observer (CF) using a standardized procedure to identify possible labeling errors. Labeling quality was judged sufficient in 1705 cases that were retained for the sulcal morphometry analysis. For each identified sulcus, we computed the following morphological measures: surface of the sulcus medial sheet, maximal and mean depth along the sulcus, sulcal width, (defined as the mean distance between the two walls of the pial surface), and gray matter thickness in the neighborhood of the sulcus.

### Statistical analysis

Based on the global criterion, we computed the proportions of IHI within the population along with confidence intervals at 95%. We compared the proportion of IHI between left and right hemispheres, between males and females and between left-handed and right-handed subjects using χ^2^ tests. For all individual criteria C1 to C5, we computed the frequencies of the different possible grades (from 0 to 2). Results between left and right hippocampi were compared using χ^2^ tests. Finally, we studied the relationship between the sum of individual criteria (called IHI score in the following) and the global criterion C0 and estimated an optimal threshold on the IHI score to classify IHI, using C0 as a reference. This allowed dividing the population into two groups for each side denoted as IHI (subjects with IHI) and non-IHI (subjects without IHI in any side).

To explore whether subjects with IHI exhibit atypical anatomical patterns outside of the hippocampus, we compared the sulcal characteristics between IHI and non-IHI groups using Student's *t*-test. Corresponding effect sizes were estimated using Cohen's d coefficient. We assessed both ipsilateral (i.e., left hippocampus with left sulci and right hippocampus with right sulci) and contralateral (i.e., left hippocampus with right sulci and right hippocampus with left sulci) associations. *P*-values were corrected for multiple comparisons using Bonferroni correction (45 sulci × 5 measures × 4 associations = 900 tests).

## Results

### Intra- and inter-observer reproducibility

Results of kappa tests for the intra- and inter- observer reproducibility are given in Table 5. A kappa value over 0.6 indicates a substantial agreement, and over 0.8 a very strong agreement (Viera and Garrett, 2005). In all cases, intra- and inter-observer agreements were beyond substantial (0.6). Very strong agreements (over 0.8) were observed in the vast majority of cases (14/20).

**Table 5:** **Intra- and inter-observer reproducibility of the different criteria**.

	**C0**	**C1**	**C2**	**C3**	**C5**
CC1 vs. CC2	0.80	0.74	0.78	0.81	0.73
	CI: [0.66; 0.95]	CI: [0.63; 0.86]	CI: [0.68; 0.89]	CI: [0.71; 0.90]	CI: [0.58; 0.88]
FC1 vs. FC2	0.89	0.71	0.82	0.87	0.87
	CI: [0.78; 0.99]	CI: [0.59; 0.83]	CI: [0.70; 0.93]	CI: [0.76; 0.92]	CI: [0.76; 0.98]
CC1 vs. FC1	0.79	0.64	0.81	0.86	0.86
	CI: [0.63; 0.94]	CI: [0.52;0.76]	CI: [0.72; 0.91]	CI: [0.78; 0.94]	CI: [0.75; 0.97]
CC2 vs. FC2	0.87	0.82	0.88	0.87	0.80
	CI: [0.75; 0.99]	CI: [0.72; 0.92]	CI: [0.81; 0.96]	CI: [0.80; 0.95]	CI: [0.66; 0.94]

CC1, first evaluation of observer CC; CC2, second evaluation of the observer CC; FC1, first evaluation of observer FC; FC2, second evaluation of the observer FC; CI, Confidence Interval at 95%. Kappa tests for C0 and C5. Weighted kappa tests for C1, C2, and C3. Confidence intervals (CI) are at 95%.

### Results of visual evaluation of IHI

Table 6 presents the prevalence of IHI according to the global criterion C0. Total IHI was found in 17% of normal subjects for the left hippocampus and 6% for the right. IHI were significantly more frequent for the left hippocampus compared to the right (χ^2^ test, χ^2^ = 129.2, *DF* = 2, *p* < 10^−28^). Table 7 displays the co-occurrences of left and right IHI. One can note that the majority of right IHI are in fact bilateral IHI, unilateral right IHI having low frequency. On the other hand, unilateral left IHI are common.

**Table 6:** **Frequency (in % of each side) of IHI, according to the global criterion C0, for left and right hippocampi**.

**C0**	**No IHI**	**Partial IHI**	**IHI**
Left	70.9%	11.9%	17.1%
	CI: [68.9%; 72.9%]	CI: [10.5%; 13.3%]	CI: [15.5%; 18.7%]
Right	84.6%	9.0%	6.5%
	CI: [83.0%; 86.2%]	CI: [7.7%; 10.3%]	CI: [5.4%; 7.6%]

CI, Confidence Interval at 95%.

**Table 7:** **Co-occurences (in % of the population) of IHI for the left and right hippocampi, according to the global criterion C0**.

**Left vs. Right**	**No IHI Right**	**Partial IHI Right**	**IHI Right**
No IHI Left	65.9%	3.1%	1.9%
	CI: [63.8%; 68.0%]	CI: [2.3%; 3.9%]	CI: [1.3%; 2.5%]
Partial IHI Left	7.9%	3.5%	0.5%
	CI: [6.7%; 9.1%]	CI: [2.7%; 4.3%]	CI: [0.2%; 0.8%]
IHI Left	10.8%	2.3%	4.0%
	CI: [9.4%; 12.2%]	CI: [1.6%; 3.0%]	CI: [3.1%; 4.9%]

CI, Confidence Interval at 95%.

The frequencies did not differ between males and females for criterion C0 (χ^2^ = 4.41, *DF* = 2, *p* = 0.11 for left; χ^2^ = 1.29, *DF* = 2, *p* = 0.52 for right). The frequencies also did not depend on handedness (χ^2^ = 2.29, *DF* = 2, *p* = 0.89 for left; χ^2^ = 5.07, *DF* = 2, *p* = 0.54 for right).

**Figure 4:**
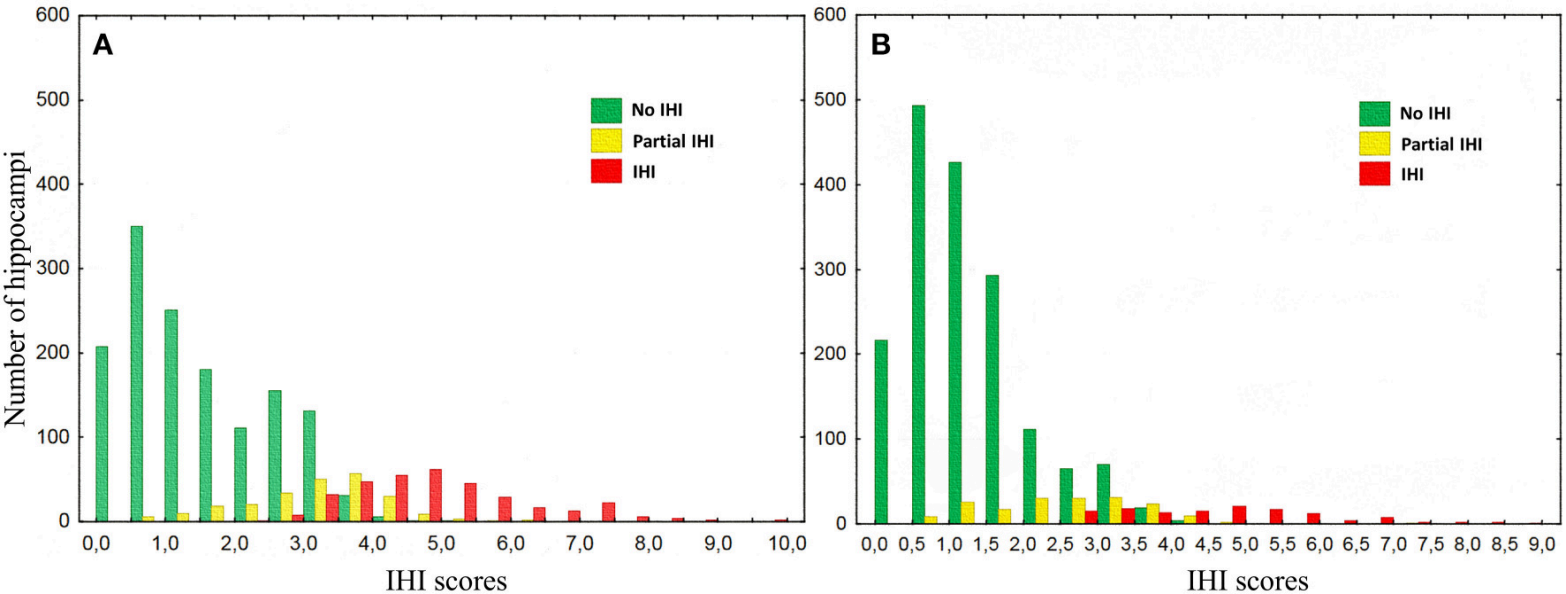
**Histograms of the sum of grades of individual criteria categorized by the global criterion C0, for left (A) and right (B) hippocampi**.

For all individual criteria, the repartition was statistically different between left and right (Table 8). The sum of grades for all individual criteria (C1 to C5) provides an overall degree of IHI between 0 and 10, denoted as IHI score. Figure 4 shows the repartition of IHI score with respect to the grade of the global criterion C0. We can note that the populations with Total IHI and without IHI are well separated. On the other hand, the intermediate class of “Partial IHI” overlaps with the two others. This highlights the consistency between the global criterion C0 and the individual criteria C1 to C5.

**Table 8:** **Repartition of grades for each individual criterion (in % of each criteria Ci)**.

	**Left**					**Right**					
	**0**	**0.5**	**1**	**0.5**	**2**	**0**	**0.5**	**1**	**1.5**	**2**	***p*-value**
C1	23%	44%	23%	8%	1%	20%	28%	18%	3%	0%	*p* < 10^−26^
C2	18%	42%	28%	11%	1%	9%	57%	29%	4%	0%	*p* < 10^−81^
C3	27%	40%	21%	10%	2%	38%	39%	17%	5%	1%	*p* < 10^−20^
C4	97%	NA	NA	NA	3%	98%	NA	NA	NA	2%	*p* < 10^−4^
C5	59%	NA	20%	NA	20%	85%	NA	6%	NA	9%	*p* < 10^−73^

NA, Not Applicable. We tested whether the repartition differs between left and right for each criterion (χ^2^ test).

Furthermore, we computed the optimal threshold grade on IHI score to classify a given hippocampus into IHI or not, using the global criterion C0 as a reference. To compute this threshold, we used only hippocampi with a C0 grade of 0 (absence of IHI) or 2 (Total IHI). We then computed the threshold on IHI score that maximizes the accuracy of the classification between cases with and without IHI. The optimal threshold is 3.75, i.e., hippocampi without IHI correspond to IHI score < 4, and hippocampi with IHI correspond to IHI score ≥4. Table 9 reports the frequencies of IHI using this threshold, it indicates both frequencies obtained without taking into account the hippocampi with C0 = 1 (which are thus very close to those reported in Table 6) and frequencies obtained when classifying all hippocampi.

**Table 9:** **Frequency (in % of the whole population for each side) of IHI using the threshold IHI score <4**.

	**Left hippocampi**		**Right hippocampi**	
	**Fixing the partial IHI**	**Classifying all hippocampi**	**Fixing the partial IHI**	**Classifying all hippocampi**
IHI	17%	22%	6%	8%
No IHI	71%	78%	85%	92%

By fixing the partial IHI group (i.e., we ignore this group for the classification), or by classifying the whole population.

### Sulcal morphometry

Sulci extraction was considered of sufficient quality for 1705 subjects. To ensure that restriction to this subpopulation did not bias the results, we computed IHI frequencies in these 1705 subjects. For left hippocampi, 383 were part of the IHI group and 1265 of the non-IHI group. For right hippocampi, 134 were part of the IHI group and 1265 of the non-IHI group. These proportions are similar to those of the whole population (presented in Table 9, second and fourth columns).

**Figure 5:**
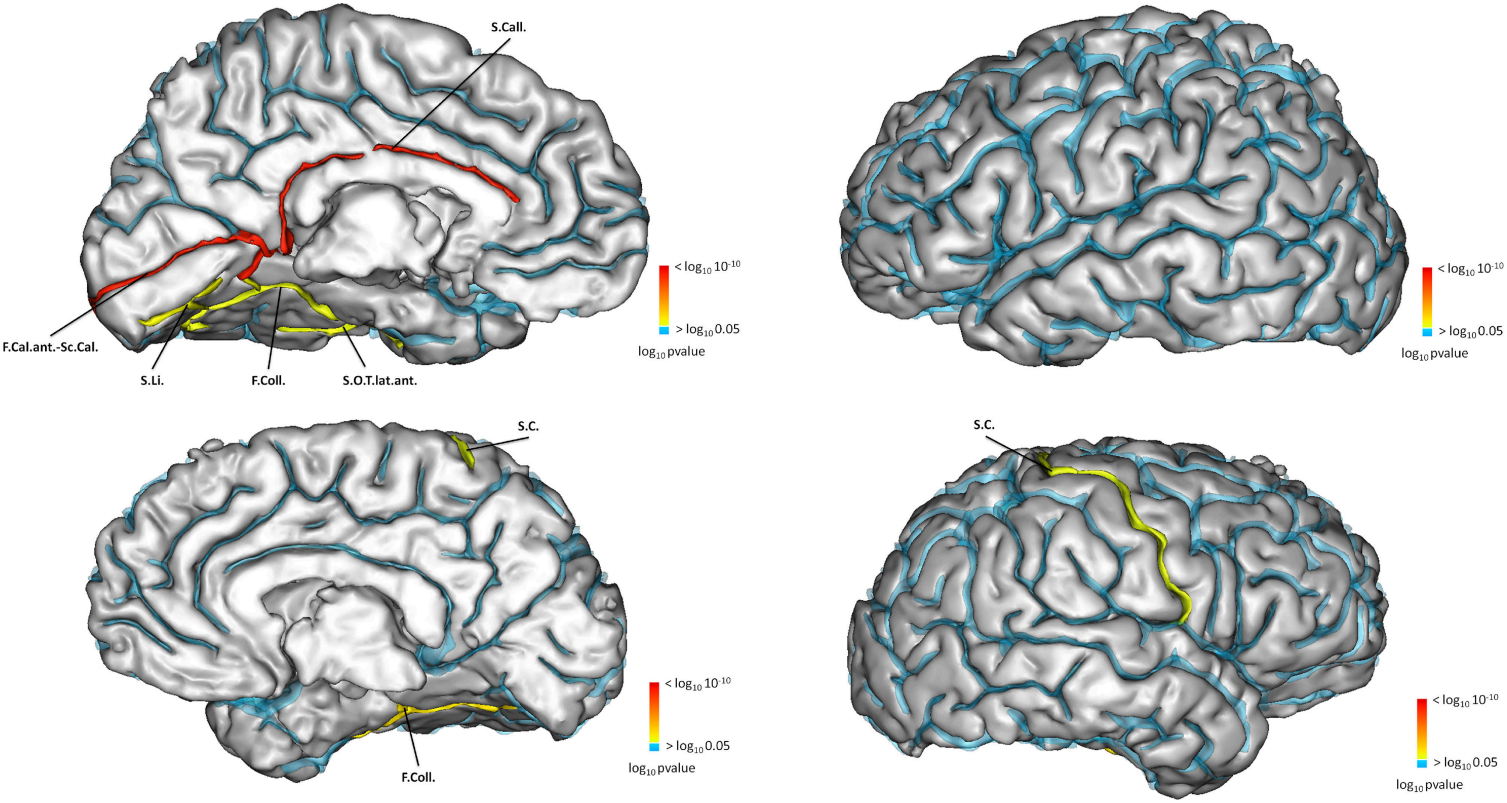
**Sulci of the left hemisphere (top) significantly different for left hippocampi with or without IHI**. Sulci of the right hemisphere (bottom) significantly different between right hippocampi with or without IHI. The color map indicates the value of the corrected *p*-value. *p* > 0.05 are in blue.

Sulcal characteristics that were significantly different between the two groups are reported in Table 10 and displayed on Figure 5. Differences in left side were ipsilateral to the IHI, and in both sides for the right side.

**Table 10:** **Results of association between IHI scores and sulci measurements**.

	**Sulci**	**Measure**	**Mean of IHI group**	**Mean of no-IHI group**	**Cohen's d**	***T***	**Corrected *p*-value**
Left Hippocampi	Calcarine fissure (F.Cal.)	GM thickness	3.7875	3.8853	−0.33	−5.72	1.13e-05
vs. Left		Opening	1.8468	1.5848	0.5	9.72	8.79e-19
Hemisphere		Surface	2127.1	2362.0	−0.36	−5.89	4.11e-06
	Collateral sulcus (F.Coll.)	Max depth	26.560	21.384	0.36	7.19	8.52e-10
		Opening	1.6391	1.5341	0.29	5.13	2.9e-04
	Callosal sulcus (S.Call.)	Max depth	11.263	12.520	−0.38	−6.41	1.63e-07
		Mean depth	6.7289	7.2136	−0.45	−7.7	2.10e-11
		Length	100.59	111.48	−0.31	−5.5	4.02e-05
		Opening	4.1009	3.5540	0.38	6.9	6.32e-09
		Surface	932.01	1113.0	−0.42	−7.28	4.65e-10
	Lingual sulcus (S.Li.)	Mean depth	10.014	9.5270	0.24	4.37	1.17e-02
	Occipito-temporal sulcus (S.O.T.lat.ant)	Opening	3.1394	2.8511	0.22	4.12	3.62e-02
Right Hipp	Collateral sulcus (F.Coll.)	Max depth	26.205	21.436	0.35	5.05	4.61e-04
vs. Right Hem	Central sulcus (S.C.)	GM thickness	3.6417	3.7501	−0.32	−4.08	4.25e-02
Right Hipp vs. Left Hem	Calcarine fissure (F.Cal.)	Opening	1.8009	1.5848	0.46	5.72	1.17e-05

GM, gray matter; Hipp, Hippocampi; Hem, Hemisphere; T, T Statistic of the t-test; d, effect size. The table lists the sulci for which a significant difference between the two groups was found (statistical threshold was p < 0.05 corrected for multiple comparisons using Bonferroni correction).

## Discussion

In this paper, we characterized IHIs and studied their prevalence in a large population of young normal subjects. We demonstrated that IHI are a common anatomical pattern in normal subjects, that they are much more frequent in the left hemisphere and that they are associated to more widespread morphological changes outside the hippocampus.

The existence of IHI in normal subjects was already known (Bernasconi et al., 2005; Bajic et al., 2009; Gamss et al., 2009) but their prevalence was a matter of debate, some authors arguing that IHI are a rare finding in patients without epilepsy (Gamss et al., 2009) and others reporting that IHI are a common variant (Bajic et al., 2009; Raininko and Bajic, 2010). The discrepancies between previous studies can be due to: (1) relatively small number of subjects resulting in imprecise estimates of the frequency; (2) populations that mixed healthy controls and patients without epilepsy but with other neurological conditions; (3) different sets of criteria for assessing IHI. Our study relied on a large population of normal subjects, providing reliable estimates with narrow confidence intervals. Furthermore, we included only young normal subjects thus avoiding the occurrence of medical conditions that could bias the estimates or of age-related morphological changes that could make the visual evaluation difficult.

Incomplete inversions were clearly more frequent in the left than in the right hemisphere. Furthermore, unilateral right IHI were particularly rare. This finding is consistent with previous studies (Barsi et al., 2000; Bajic et al., 2009; Raininko and Bajic, 2010). It seems that an asymmetric development of the hippocampus is common, and that this asymmetry is lateralized, the right hippocampus developing at faster pace in a vast majority of cases (Bajic et al., 2012). This implies that the hippocampal inversion as well as the closing of the hippocampal sulcus may occur earlier in the right hemisphere. One can thus think that, if the hippocampal inversion process is stopped at a specific time, it may be incomplete only in the left hemisphere. Furthermore, in normal adults, various studies have shown asymmetry in hippocampal volumes, the right being larger (Pedraza et al., 2004; Lucarelli et al., 2013). Whether this volumetric asymmetry could be related to increased prevalence of IHI in the left hippocampus remains to be studied. Furthermore, there are also functional differences between the two hippocampi: the right is predominantly involved in memory for locations within an environment whereas the left hippocampus plays a central role in context-dependant episodic memory or in autobiographical memory (Bohbot et al., 1998; Maguire, 2001; Burgess, 2002). Asymmetry of gene expression levels has been demonstrated in the hippocampi of rats (Moskal et al., 2006) as well as the human cerebral cortex (Sun et al., 2005), which could in turn provide a basis of structural and functional asymmetries.

Compared to subjects without IHI, subjects with IHI had different morphological characteristics in several cortical sulci. This demonstrates that morphological changes associated with IHI are not confined to the hippocampus or to the medial temporal lobe. In left IHI, sulcal changes were located on the internal part of the cortex (Figure 5), and followed the limbic lobe which is involved in memories formation, long term memory and emotions, and includes the hippocampus (Duvernoy, 2005). In right IHI, differences were less extensive and confined to the collateral sulcus and the central sulcus of the right hemisphere. For the right IHI we also found differences in the left hemisphere, however right IHI are mainly bilateral; indeed in Table 10, the results for the calcarine fissure are very similar for left IHI vs. left hemisphere and right IHI vs. left hemisphere. Therefore, we cannot say that right IHI are related with morphological changes in the left hemisphere.

The hippocampal formation is the first cortical area to differentiate (Humphrey, 1967) and at 30 gestational weeks (GW), the hippocampus formation has acquired most of the features observed in the adult population. Primordial hippocampi seem to be observable from 7 GW (Baker and Barkovich, 1992). At 10 GW, the dentate gyrus and the cornu Ammonis are rudimentary structures situated in the postero-medial wall of the lateral ventricles (Humphrey, 1967). At 13 GW, the hippocampus goes from the frontal lobe to the temporal lobe on the postero-medial wall of the lateral ventricles, and surrounds a widely open hippocampal sulcus (Humphrey, 1967; Kier et al., 1997). At 16 GW the hippocampus reduces in size (relatively to the size of the brain which increases), pushed by the growth of the corpus callosum and therefore has to leave the frontal lobe to only occupy the temporal lobe. Furthermore, this growth of the corpus callosum coincides with the growth of the callosal sulcus which appears around the 16th GW (Chi et al., 1977; Larroche, 1981; Nishikuni and Ribas, 2012). The other significant sulci found in the study appear after, during the inversion phase of the hippocampus, which occurs between the 20th GW and the 30th GW (Bajic et al., 2010). The next to appear is the calcarine fissure, around the 20th GW (Chi et al., 1977; Dorovini-Zis and Dolman, 1977; Nishikuni and Ribas, 2012). The collateral sulcus appears around the 24th GW (Chi et al., 1977; Garel et al., 2001; Nishikuni and Ribas, 2012). In our study, morphological characteristics of these sulci were altered in subjects with IHI.

Different criteria have been used in the literature to assess IHI, making it difficult to compare results across studies (Gamss et al., 2009; Raininko and Bajic, 2010). Moreover, these visual scales differ in terms of number of features to assess; those with many features being not easily applicable to larger series of over 1000 subjects. In this work, we adapted existing criteria from the literature in order to propose a new visual scale of IHI that includes the most representative published criteria of IHI (Baulac et al., 1998; Bernasconi et al., 2005), includes a reasonable number of items and leads to a robust assessment. We demonstrated that our criteria are highly reproducible across observers and rating sessions. We also defined a global criterion assessing the overall presence of an IHI. Although this criterion could theoretically be more subjective, we showed that its reproducibility is as good as for the other criteria. Furthermore, the global criterion was highly consistent with the individual scores. The detailed criteria presented above could lead to more comparable results across studies. The distribution of the sum of individual criteria indicates that there is a continuum between a normal hippocampus and IHI, with various intermediate degrees of hippocampal inversions. The sum of individual criteria can be used to assess the degree of IHI of hippocampi and for subsequent correlation with other neuroanatomical or behavioral features. It is also possible to use the global criterion in order to propose a threshold on the sum of criteria to obtain a binary classification into IHI and typical hippocampi.

Compared to the other criteria, there were much less subjects with an abnormal score for criterion C4 (about 3% of subjects), corresponding to a thicker subiculum. Interestingly, in Bernasconi et al. (2005), none of the 50 healthy subjects had an abnormally thick subiculum against 17 of the 76 patients with MCD, and 4 of the 30 patients with TLE. This criterion might thus be overrepresented in patients with MCD or TLE. Nevertheless, this hypothesis would need to be further tested in a larger population of patients with epilepsy or MCD.

IHI are highly prevalent in patients with epilepsy (30–50%), in particular in patients with MCD but also in TLE (Lehéricy et al., 1995; Baulac et al., 1998; Bernasconi et al., 2005; Bajic et al., 2009). IHI have also been described in patients with agenesis of the corpus callosum (Atlas et al., 1986), and patients with genetic anomalies (Fitoz et al., 2003; Grosso et al., 2003; Andrade et al., 2013; Boronat et al., 2015) associated with neuropsychiatric disorders including autism spectrum disorders (Campbell et al., 2006) and schizophrenia (Baker et al., 2011). IHI are thus likely to be a marker of more extensive atypical development that may render the brain more vulnerable to pathological processes. Nevertheless, further studies are needed to fully describe IHI in different neurological and psychiatric disorders and to elucidate their putative role in pathogenesis. By providing an extensive characterization of IHI in the general population, our study shall provide a reference for future research on the role of IHI in different pathological conditions.

Our study has the following limitations. We applied a strict Bonferroni correction to the sulcal morphometry analysis. This procedure has the advantage to strictly control for false positives. Nevertheless, it may be overly conservative since sulcal measures are not statistically independent. Effect sizes were small to moderate but were within the typical range of morphometric studies of brain development, as for example these studies (Haar et al., 2014; Klein et al., 2014; Lefebvre et al., 2015) that found significant differences in brain structures volume with small to moderate effects. Furthermore, taking into account that the sulci are highly variable, we cannot expect observing bigger effects. Further studies using more comprehensive models of sulcal shapes are needed to clarify the nature of the relationships between IHI and sulcal changes.

In conclusion, IHIs are frequently found in normal subjects, predominantly in the left hemisphere. IHI are associated with extra-hippocampal morphological changes, in particular in sulci of the limbic lobe.

## Author contributions

Guarantor of integrity of entire study, CC; study concepts and design or data acquisition or data analysis and interpretation, all authors; manuscript drafting or manuscript revision for important intellectual content, all authors; approval of final version of submitted manuscript, all authors; literature research, CC, FC, OC, DH; clinical studies, TB, AB, UB, CB, AC, PC, HF, JG, HG, PG, AH, BI, HL, J-LM, FN, MP, DO, TP, LP, MS, HW, RW, VF, GS; statistical analysis, CC, RT, FC, JS, CF, AM, J-FM, JAG, OC; and manuscript editing, all authors.

## Source of funding

This work was supported by ANR (project HM-TC, grant number ANR-09-EMER-006), by the CATI project (Fondation Plan Alzheimer), and by the program “Investissements d'avenir” (grant number ANR-10-IAIHU-06). IMAGEN was supported by the European Union-funded FP6 (LSHM-CT-2007-037286), the FP7 projects IMAGEMEND (602450) and MATRICS (603016), and the Innovative Medicine Initiative Project EU-AIMS (115300-2), Medical Research Council Program Grant “Developmental pathways into adolescent substance abuse” (93558), the NIHR Biomedical Research Centre “Mental Health” as well as the Swedish funding agency FORMAS. Further support was provided by the Bundesministerium für Bildung und Forschung eMED SysAlc: 01ZX1311E (Berlin); AERIAL: 01EE1406A (Berlin).

### Conflict of interest statement

The authors declare that the research was conducted in the absence of any commercial or financial relationships that could be construed as a potential conflict of interest.

## Footnotes

^1^London: Psychiatry, Nursing and Midwifery (PNM) Research Ethics Subcommittee (RESC), Waterloo Campus, King's College London. Nottingham: University of Nottingham Medical School Ethics Committee. Mannheim: Medizinische Fakultaet Mannheim, Ruprecht Karl Universitaet Heidelberg and Ethik-Kommission II an der Fakultaet fuer Kliniksche Medizin Mannheim. Dresden: Ethikkommission der Medizinischen Fakultaet Carl Gustav Carus, TU Dresden Medizinische Fakultaet. Hamburg: Ethics board, Hamburg Chamber of Phsyicians. Paris: CPP IDF VII (Comité de protection des personnes Ile de France), ID RCB: 2007-A00778-45 September 24th 2007. Dublin: TCD School of Psychology REC. Berlin: ethics committee of the Faculty of Psychology. And Mannheim's ethics committee approved the whole study.
